# Assessing Changes in Quality of Life Measures, Resilience, and Personal Recovery, Pre- and Post-Discharge from Inpatient Mental Health Units in Alberta: Analysis of Control Group Data from a Randomized Trial.

**DOI:** 10.1192/j.eurpsy.2024.701

**Published:** 2024-08-27

**Authors:** E. Owusu, R. Shalaby, W. Mao, H. Elgendy, N. Shalaby, B. Agyapong, A. Nichols, E. Eboreime, M. A. Lawal, N. Nkire, V. I. Agyapong

**Affiliations:** ^1^Psychiatry, University of Alberta, Edmonton; ^2^Alberta Health Services, Queen Elizabeth II Hospital, Grande Prarie; ^3^Psychiatry, Dalhousie University, Halifax, Canada

## Abstract

**Introduction:**

The transition from hospital to community settings for most mental health service users is often hindered by challenges that affect community adjustment and continuity of care. The first few weeks and days after discharge from mental health inpatient units represent a critical phase for many service users.

**Objectives:**

This paper aims to evaluate the changes in quality of Life status, resilience, and personal recovery of individuals with mental health challenges recently discharged from acute mental health care into the community.

**Methods:**

Data for this study were collected as part of a pragmatic stepped-wedge cluster-randomized, longitudinal approach in Alberta. A paired sample t-test and Chi-squared/Fisher test were deployed to assess changes from baseline to six weeks in the recovery assessment scale (RAS), brief resilience scale (BRS), and EuroQol-5d (EQ-5D), using an online questionnaire.

**Results:**

A total of 306 service users were recruited, and 88 completed both baseline and six weeks, giving a response rate of 28.8%. There was no statistically significant change in the level of resilience, recovery and quality of life as measured with the brief resilience scale, recovery assessment scale and EQ-5D from baseline to six weeks (*p* > 0.05).

**Conclusions:**

The study showed that there was neither an improvement nor deterioration in resilience, recovery, or quality of life status of service users six weeks post-discharge from inpatient mental health care. The lack of further progress calls into question whether the support available in the community when patients leave inpatient care is adequate to promote full recovery.

**Disclosure of Interest:**

None Declared

